# Some Account of a Peculiar Species of Plague Which Depopulated West Barbary in 1799 and 1800, and to the Effects of Which the Author Was an Eye-Witness
*See Travels in Africa by the above gentleman.


**Published:** 1809-09

**Authors:** James Grey Jackson

**Affiliations:** Consul at Mogodor


*Some Account of a peculiar Species of Plague which depopulated West Barbary in* 1799 *and* 1800, *and to the Effects of which the Author was an Eye-witness.* By* JAMES GREY JACKSON, Esq. Consul at Mogodor.

FROM various circumstances and appearances, and from the character of the epidemical distemper which raged lately in the South of Spain, there is every reason to suppose it was similar to that distemper or plague which depopulated West Barbary; for whether we call it by the more reconcilable appellation of the epidemy, or yellow fever, it was undoubtedly a plague, and a most destructive one, for wherever it prevailed, it invariably carried off, in a few months, one-half, or one-third, of the population.

It does not appear how the plague originated in Faz in the year 1799. Some persons who were there at the time it broke out, have confidently ascribed it to infected merchandize imported into that place from the East; whilst others, of equal veracity and judgment, have not scrupled to ascribe it to the locusts which had infested West Barbary during the seven preceding years, the destruction of which was followed by the (jedrie) small-pox, which pervaded the country, and was generally fatal. The jedrie is supposed to be the forerunner of this species of epidemy, as appears by an ancient Arabic manuscript, which gives an account of the same disorder having carried off two-thirds of the inhabitants of West Barbary about four centuries since. But however this destructive epidemy originated, its leading features were novel, and its consequences more dreadful, than the common plague of Turkey, or that of Syria, or Egypt, as will appear by the following observations.

In the month of April, 1799, a plague of the most destructive nature manifested itself in the city of Old Faz, which soon after communicated itself to the new city, carrying off one or two the first day, three or four the second day, six or eight the third day, and increasing progressively, until the mortality amounted to two in the hundred of the aggregate population, continuing *with unabated violence*, ten, fifteen, or twenty days; being of longer duration in old than in new towns; then diminishing in a progressive proportion from one thousand a day, to nine hundred, then to eight hundred, and so on until it disappeared.

* See Travels in Africa by the above gentleman.

Whilst it raged in the town of Mogodor, a small village (Diabet), situated about two miles south-east of that place, remained uninfected, although the communication was open between them: on the thirty-fourth day, however, after its first appearance at Mogodor, this village was discovered to be infected, and the disorder raged with great violence, making dreadful havock among the human species for twenty-one days, carrying off, during that period, one hundred persons out of one hundred and thirty-three, the original population of the village before the plague visited it; none died after this, and those who were infected, recovered in the course of a month or two, some losing an eye, or the use of a leg or an arm.

Many similar circumstances might be here adduced relative to the numerous and populous villages dispersed through the extensive Shelluh province of Haha, all which shared a similar or a worse fate. Travelling through this province shortly after the plague had exhausted itself, I saw many uninhabited ruins, which I had before witnessed as flourishing villages; on making inquiry concerning the population of these dismal remains, I was informed that in one village, which contained six hundred inhabitants, four persons only had escaped the ravage. Other villages, which had contained four or five hundred, had only seven or eight survivors left to relate the calamities they had suffered. Families which had retired to the country to avoid the infection, on returning to town, when all infection had apparently ceased, were generally attacked, and died; a singular instance of this kind happened at Mogodor, where, after the mortality had subsided, a corps of troops arrived from the city of Terodant, in the province of Suse, where the plague had been raging, and had subsided; these troops, after remaining three days at Mogodor, were attacked with the disease, and it raged exclusively among them for about a month, during which it carried off two-thirds of their original number, one hundred men; during this interval the other inhabitants of the town were exempt from the disorder, though these troops were not confined to any particular quarter, many of them having had apartments in the houses of the inhabitants of the town.

The destruction of the human species in the province of Suse was considerably greater than elsewhere; Terodant, formerly the metropolis of a kingdom, but now that of Suse, lost, when the infection was at its height, about eight hundred each day; the ruined, but still extensive and and populous city of Marocco, lost one thousand each day; the populous cities of Old and New Faz diminished in population twelve or fifteen hundred each day,* insomuch, that in these extensive cities, the mortality was so great, that the living having not time to bury the dead, the bodies were deposited or thrown altogether, into large holes, which, when nearly full, were covered over with earth. Young, healthy, and robust persons of full stamina, were for the most part attacked first, then women and children, and lastly, thin, sickly, emaciated, and old people.

After this deadly calamity had subsided, we beheld a general alteration in the fortunes and circumstances of men; we saw persons who before the plague, were common labourers, now in possession of thousands, and keeping horses without knowing how to ride them. Parties of this description were met wherever we went, and the men of family called them in derision (el wurata) the inheritors.† Provisions also became extremely cheap and abundant; the flocks and herds had been left in the fields, and there was now no one to own them; and the propensity to plunder, so notoriously attached to the character of the Arab, as well as to the Shelluh and Moor, was superceded by a conscientious regard to justice, originating from a continual apprehension of dissolution, and that the El khere,‡ as the plague was now called, was a judgment of the Omnipotent on the disobedience of man, and that it behoved every individual to amend his conduct, as a preparation to his departure for paradise.

The expense of labour at the same time increased enormously, and never was equality in the human species more conspcicuous than at this time; when corn was to be ground, or bread baked, both were performed in the houses of the affluent, and prepared by themselves, for the very few people whom the plague had spared, were insufficient to administer to the wants of the rich and independant, and they were accordingly compelled to work for themselves, performing personally the menial offices of their respective families.

The country being now depopulated, and much of the territory without owners, vast tribes of Arabs emigrated from

* There died, during the whole of the above periods, in Marocco, 50,000; in Faz, 65,000; in Mogodor, 4,500; and in Saffy, 5,000; in all 124,500 souls!

† Des gens parvenues, as the French express it; or upstarts.

‡ The good, or benediction.

from their abodes in the interior of Sahara, and took possession of the country contiguous to the river Draha, as well as many districts in Suse; and, in short, settling themselves, and pitching their tents wherever they found a fertile country with little or no population.

The symptoms of this plague varied in different patients; the variety of age and constitution gave it a like variety of appearance and character; in some it manifested itself by a sudden and violent shivering, in others by a sudden delirium, succeeded by great and unquenchionable thirst. Cold water was eagerly resorted to by the unwary and imprudent, and proved fatal to those who indulged in its momentary relief. Some had one, two, or more buboes, which formed themselves, and became often as large as a walnut, in the course of a day; others had a similar number of carbuncles: others had both buboes and carbuncles, which generally appeared in the groin, under the arm, or near the breast. Those who were affected * with a shivering, having no buboe, carbuncle, spots, or any other exterior disfiguration, were invariably carried off in less than twenty-four hours, and the body of the deceased became quickly putrified, so that it was indispensibly necessary to bury it a few hours after dissolution. I recommended Mr. Baldwin's † invaluable remedy of olive oil, applied according to his directions; several Jews, and some Mooselmin, were induced to try it, and I was afterwards visited by many, to whom I had recommended it, and had given them written directions in Arabic how to apply it; and I do

* M'drob is an idiom in the Arabic language somewhat difficult to render into English; it is well known that the Mohammedans are predestinarians, and that they believe in the existence of spirits, devils, &c.; their idea of the plague is, that it is a good or blessing sent from God to clear the world of a superfluous population—that no medicine or precaution can cure or prevent it; that every one who is to be a victim to it is (mktube) recorded in the Book of Fate; that there are certain Genii who preside over the fate of men, and who sometimes discover themselves in various forms, having often legs similar to those of fowls; that these Genii are armed with arrows; that when a person is attacked by the plague, which is called in Arabic l'amer, or the destiny or decree, he is shot by one of these Genii, and the sensation of the invisible wound is similar to that from a musquet-ball; hence the universal application of M'drob to a person afflicted with the plague, i. e. he is shot; and if he die, ufah, ameruh, his destiny is completed or terminated, (in this world.) I scarcely ever yet saw the Mooselmin who did not affirm that he had at some time of his life seen these Genii, and they often appear, they say, in rivers.

† Late British Consul in Egypt.

do not know any instance of its failing when persevered in, even after the infection had manifested itself.

I have no doubt but the epidemy which made its appearance at Cadiz, and all along the southern shores of Spain, immediately as the plague was subsiding in West Barbary, was the same disorder with the one above described, suffering, after its passage to a Christian country, some variation, originating from the different modes of living, and other circumstances; for nothing can be more opposite than the food, dress, customs, and manners of Mohammedans and Christians, notwithstanding the approximation of Spain to Marocco. We have been credibly informed, that it was communicated originally to Spain by two infected persons who went from Tangier to Estapona, a small village on the opposite shore; who, after eluding the vigilance of the guards, reached Cadiz. We have also been assured that it was communicated by some infected persons who landed in Spain, from a vessel that had loaded produce at L'araiche in West Barbary. Another account was, that a Spanish privateer, which had occasion to land its crew for the purpose of procuring water in some part of West Barbary, caught the infection from communicating with the natives, and afterwards proceeding to Cadiz, spread it in that town and the adjacent country.

It should be observed, for the information of those who may be desirous of investigating the nature of this extraordinary distemper, that, from its character and its symptoms, approximating to the peculiar plague, which (according to the before mentioned Arabic record) ravaged and depopulated West Barbary four centuries since, the Arabs and Moors were of opinion it would subside after the first year, and not appear again the next, as the Egyptian plague does; and agreeably to this opinion, it did not re-appear the second year; neither did St. John's day, or that season, affect its virulence; but about that period there prevails along the coast of West Barbary a trade wind, which beginning to blow in the month of May, continues throughout the months of June, July, and August, with little intermission. It was apprehended that the influence of this trade wind, added to the superstitious opinion of the plague ceasing on St. John's day, would stop, or at least sensibly diminish the mortality; but no such thing happened: the wind did set in, as it invariably does about St. John's day; the disorder, however, increased at that period, rather than diminished. Some persons were of opinion, that the infection maintained its virulence till the last; that the decrease of mortality did not originate from a decrease of the *miasma,* but from a decrease of population, and a consequent want of subjects to prey upon; and this indeed is a plausible idea; but admitting it to be just, how are we to account for the almost invariable fatality of the disorder, when at its height, and the comparative innocence of it, when on the decline? for *then,* the chance to those who had it, was, that they would recover and survive the malady.

The old men seemed to indulge in a superstitious tradition, that when this peculiar kind of epidemy attacks a country, it does not return or continue for three or more years, but disappears altogether (after the first year), and is followed the seventh year by contagious rheums and expectoration, the violence of which lasts from three to seven days, but is not fatal. Whether this opinion be in general founded in truth I cannot determine; but in the spring of the year 1806, which was the seventh year from the appearance of the plague at Faz in 1799, a species of influenza pervaded the whole country; the patient going to bed well, and on rising in the morning, a thick phlegm was expectorated, accompanied by a distressing rheum, or cold in the head, with a cough, which quickly reduced those affected to extreme weakness, but was seldom fatal, continuing from three to seven days, with more or less violence, and then gradually disappearing.

During the plague at Mogodor, the European merchants shut themselves up in their respective houses, as is the practice in the Levant; I did not take this precaution, but occasionally rode out to take exercise on horseback. Riding one day out of the town, I met the Governor's brother, who asked me where I was going, when every other European was shut up? "To the garden," I answered. "And are you not aware that the garden and the adjacent country is full of (Genii) departed souls, who are busy in smiting with the plague every one they meet?" I could not help smiling, but told him, that I trusted to God only, who would not allow any of the Genii to smite me unless it were his sovereign will, and that if it were, he could effect it without the aid of Genii. On my return to town in the evening, the sandy beach from the town-gate to the sanctuary of Seedi Mogodole * was covered with biers. My daily observations convinced me that the epidemy

* A sanctuary a mile south-east of the town of Mogodor, from whence the town receives its name.

my was not caught by approach, unless that approach was accompanied by an inhaling of the breath, or by touching the infected person; I therefore had a separation made across the gallery, inside of my house, between the kitchen and dining parlour, of the width of three feet, which is sufficiently wide to prevent the inhaling the breath of a person. From this partition or table of separation I took the dishes, and after dinner returned them to the same place, suffering none of the servants to come near me; and in the office and counting-house, I had a partition made to prevent the too near approach of any person who might call on business; and this precaution I firmly believe to be all that is necessary, added to that of receiving money through vinegar, and taking care not to touch or smell infectious substances.

Fear had an extraordinary effect in disposing the body to receive the infection, and those who were subject thereto invariably caught the malady, which was for the most part fatal. At the breaking out of the plague at Mogodor, there were two medical men, an Italian and a Frenchman, the latter, a man of science, a great botanist, and of an acute discrimination; they however did not remain, but took the first opportunity of leaving the place for Teneriffe, so that the few Europeans had no expectation of any medical assistance except that of the natives. Plaisters of gum ammoniacum and the juice of the leaves of the opuntia, or kermuse ensarrah, i. e. prickly pear, were universally applied to the carbuncles, as well as the buboes, which quickly brought them to maturity: many of the people of property took copious drafts of coffee and Peruvian bark. The *vinaigre de quatre voleurs* was used by many, also camphor, smoking tobacco, or fumigations of gum Sandarac; straw was also burned by some, who were of opinion, that any thing which produced abundance of smoke was sufficient to purify the air of pestilential effluvia.

During the existence of the plague I had been in the chambers of men on their death-bed: I had had Europeans at my table, who were infected, as well as Moors, who actually had buboes on them: I took no other precaution than that of separation, carefully avoiding to touch the hand, or inhale the breath; and, notwithstanding what may have been said, I am decidedly of opinion that the plague, at least this peculiar species of it, is not produced by any infectious principle in the atmosphere, but caught solely by touching infected substances, or inhaling the

 breath breath of those who are deceased; and that it must not be confounded with the common plague of Egypt, or Constantinople, being a malady of a much more desperate and destructive kind. It has been said, by persons who have discussed the nature and character of the plague, that the cultivation of a country, the draining of the lands, and other agricultural improvements, tend to eradicate or diminish it; but at the same time we have seen countries depopulated where there was no morass or stagnate water for many days journey, nor even a tree to impede the current of air, or a town, nor any thing but encampments of Arabs, who procured water from wells of a great depth, and inhabited plains so extensive and uniform, that they resembled the sea, and are so similar in appearance after, as well as before sun-rise, that if the eye could abstract itself from the spot immediately surrounding the spectator, it could not be ascertained whether it were sea or land.

Many of the cities and towns of Marocco are visited yearly by malignant epidemies, which the natives call fruit-fevers; they originate from their indulgence in fruit, which abounds all over this fertile garden of the world. The fruits deemed most febrile are musk mellons, apricots, and all unripe stone fruits. Alpinus, de Medicina Egyptiorum, says, "Autumno grassantur febres pestilentiales multæ quæ subdole invadunt, et sæpe medicum et ægrum decipiunt."

I shall now subjoin a few cases for the further elucidation of this distemper, hoping that the medical reader will pardon any inaccuracy originating from my not being a professional man.

Case I. One afternoon I went into the kitchen, and saw the cook making bread; he appeared in good health and spirits; I afterwards went into the adjoining parlour, and took up a book to read; in half an hour the same man came to the door of the room, with his eyes starting from his head, and his bed clothes, &c. in his hands, saying, "Open the gate for me, for I am (m'drob) smitten." I was astonished at the sudden transition, and desired him to go out, and I would follow and shut the gate. The next morning he sent his wife on an errand, and got out of bed, and came to the gate half dressed, saying that he was quite recovered, and desired I would let him in. I did not, however, think it safe to admit him, but told him to go back to his house for a few days, until he should be able to ascertain that he was quite well; he accordingly returned returned to his apartments, but expired that evening, and before day-break his body was in such a deplorable state, that his feet were putrified. His wife, by attending on him, caught the infection, having a carbuncle and also buboes, and was confined two months before she recovered.

Case II. L'Hage Hamed O Bryhim, the old governor of Mogodor, had twelve or more children and four wives, who were all attacked, and died, excepting one young wife. He attended them successively to the grave; and notwithstanding that he assisted in performing the religious ceremony of washing the body, he never caught the infection. He lived some years afterwards; and out of the whole household, consisting of wives, concubines, children, and slaves, he had but one person left, which was the before mentioned young wife: this lady, however, had received the infection, and was confined some time before she recovered.

Case III. Hamed ben A——— was smitten with the plague, which he compared to the sensation of two musquet balls fired at him, one in each thigh; a giddiness and delirium succeeded, immediately afterwards a green vomiting, and he fell senseless to the ground. A short time afterwards, on the two places where he had felt as if shot, biles or buboes formed, which on suppurating, discharged a fœtid black pus: a (jimerra) carbuncle on the joint of the arm near the elbow was full of thin ichor, contained in an elevated skin, surrounded by a burning red colour. After three months confinement, being reduced to a skeleton, the disorder appeared to have exhausted itself, and he began to recover his strength, which in another month was fully re-established. It was an observation founded on daily experience, during the prevalence of this disease, that those who were attacked with a nausea at the stomach, and a subsequent vomiting of green or yellow bile, recovered after suffering in various degrees, and that those who were affected with giddiness, or delirium, followed by a discharge or vomiting of black bile, invariably died after lingering one, two, or three days, their bodies being covered with small black spots similar to grains of gunpowder: in this state, however, they possessed their intellects, and spoke rationally till their dissolution.

When the constitution was not disposed, or had not vigour enough, to throw the miasma to the surface in the form of biles, buboes, carbuncles, or blackish spots, the virulence virulence is supposed to have operated inwardly, or on the vital parts, and the patient died in less than twenty-four hours, without any exterior disfiguration.

Case IV. It was reported that the Sultan had the plague twice during the season, as many others had; so that the idea of the plague, like the small-pox, attacking a person but once in his life, is refuted. The Sultan was cured by large doses of Peruvian bark frequently repeated; and it was said that he found such benefit from it, that he advised his brothers never to travel without having a good supply. The Emperor, since the plague, always has by him a sufficient quantity of quill bark to supply his daily emergency.

Case V. H. L. was smitten with the plague, which affected him by a pain similar to that of a long needle (as he expressed himself) repeatedly plunged into his groin. In an hour or two afterwards, a (jimerra) carbuncle appeared in the groin, which continued enlarging three days; at the expiration of which period, he could neither support the pain, nor conceal his sensations. He laid himself down on a couch; an Arabian doctor applied to the carbuncles the testicles of a ram cut in half, whilst the vital warmth was still in them. The carbuncle on the third day was increased to the size of a small orange. The before mentioned remedy was daily applied during thirty days, after which he resorted to cataplasms of the juice of the (opuntia) prickly pear-tree, (feshook) gum ammoniac, and (zite el aud) oil of olives, of each one-third. This was intended to promote suppuration, which was soon effected. There remained after the suppuration a large vacuity, which was daily filled with fine hemp dipped in honey; by means of this application the wound filled up, and the whole was well in thirty-nine days.

Case VI. El H—t—e, a trading Jew of Mogodor, was sorely afflicted: he called upon me, and requested some remedy; I advised him to use oil of olives; and having Mr. Baldwin's mode of administering it,* I transcribed it in the Arabic language, and gave it to him. He followed the prescription, and assured me, about six weeks afterwards, that, with the blessing of God, he had preserved his life by that remedy only. He said, that after having been

* Mr. Baldwin observed, that whilst the plague ravaged Egypt, the dealers in oil were not affected with the epidemy, and he accordingly recommended people to anoint themselves with oil every day as a remedy.

been anointed with oil, his skin became harsh and dry, like the scales of a fish; but that in half an hour more, a profuse perspiration came on, and continued for another half hour, after which he experienced relief. This he repeated forty days, and he was then quite recovered.

Case VII. Moh—m'd ben A——— fell suddenly down in the street; he was conveyed home; three carbuncles and five buboes appeared the same day in his groin, under the joint of his knee, arm-pits, and inside the elbow; he died in three hours after the attack.

Case VIII. L. R. was suddenly smitten with this calamity whilst looking over some Marocco leather; he fell instantaneously: afterwards, when he had recovered his senses, he described the sensation as that of the pricking of needles, at every part wherein the carbuncles afterwards appeared. He died the same day in spite of medicine.

Case IX. Mr. Pacifico, a merchant, was attacked, and felt a pricking pain down the inside of the thick part of the thigh, near the sinews; he was obliged to go to bed. I visited him the next day, and was going to approach him, but he exclaimed, "Do not come near me, for although I know I have not the prevailing distemper, yet your friends, if you touch me, may persuade you otherwise, and that might alarm you; I shall, I hope, be well in a few days." I took the hint of Don Pedro de Victoria, a Spanish gentleman who was in the room, who offering me a sagar, I smoked it, and then departed; the next day the patient died. He was attended during his illness by the philanthropic Monsieur Subremont, who did not stir from his bed-side till he expired; but, after exposing himself in this manner, escaped the infection, which proceeded undoubtedly from his constantly having a pipe in his mouth.

Case X. Two of the principal Jews of the town giving themselves up, and having no hope, were willing to employ the remainder of their life in affording assistance to the dying and the dead, by washing the bodies and interring them; this business they performed during thirty or forty days, washing the bodies of those who died of the plague, and putting on them their shrouds, during which time they were not attacked. When the plague had nearly subsided, and they began to cherish hopes of surviving the calamity, they were both smitten, but after a few days illness recovered, and are now living.

From

From this last case, as well as from many others similar, but too tedious here to recapitulate, it appears that the human constitution requires a certain miasma to prepare it to receive the pestilential effluvia.

*General Observation.* When the carbuncles or buboes appeared to have a blackish rim round their base, the case of that patient was desperate, and invariably fatal. Sometimes the whole body was covered with black spots, like partridge shot; such patients always fell victims to the disorder; and those who felt the blow internally, shewing no external disfiguration, did not survive more than a few hours.

